# Arabic language version of the obsession with COVID-19 scale adaptation and validity evaluation in Saudi sample

**DOI:** 10.1186/s41983-023-00641-7

**Published:** 2023-03-28

**Authors:** Abdulaziz Alshomrani

**Affiliations:** College of Medicine, University of Bisha, Bisha, Kingdom of Saudi Arabia

**Keywords:** Obsession, Scale, COVID-19, Anxiety, Saudi

## Abstract

**Background:**

The obsession with COVID-19 scale is a reliable and validated scale developed to assess obsessions related to coronavirus infection (COVID-2019) and because of its usefulness, this paper is aiming to develop an Arabic version of the obsession with COVID-19 scale and evaluate its validity. Firstly, scale translated to Arabic through the guidelines of Sousa and Rojjanasriratw for scale translation and adaptation. Then we distributed the final version with some sociodemographic questions and an Arabic version of the COVID-19 fear scale to a convenient sample of college students. Internal consistency, factor analysis, average variable extraction, composite reliability, Pearson correlation, and mean differences has been measured.

**Results:**

Out of 253 students, 233 responded to the survey, where 44.6% of them were female. Calculated Cronbach’s alpha was 0.82, item-total correlations were 0.891–0.905, and inter-item correlations were 0.722–0.805. Factor analysis identified one factor which reflects 80.76% of the cumulative variances. The average variance extracted was 0.80, and the composite reliability was 0.95. The correlation coefficient between the two scales was 0.472.

**Conclusions:**

The Arabic version of obsession with COVID-19 scale has high values of internal consistency, and convergent validity, and has a unidimensional factor that reflects its reliability and validity.

## Background

The coronavirus infection disease (COVID-19) pandemic has become a worldwide crisis that affects almost all countries as declared by World Health Organization (WHO) in March, 2020 [1]. Many preventive measures have been recommended by disease control centers and put in place by health and governmental authorities. Social distancing, hygiene measures, face masks, limitation of traveling and movement, restrictions on public transportation, lockdowns, and curfews have been implemented fully or partially by most of the countries [2–5]. What makes this pandemic more exceptional is the long duration, high numbers of morbidities and mortalities, high detailed daily media coverage, social, economic and political consequences [6, 7].

The mental health impact of COVID-19 and its preventive measure was one of the major challenges that encounter health authorities [5, 6]. Stress-related to Covid-19 news, preventive measures, and loss of relatives, friends, or well-known people become part of the typical living day of a lot of people [5–7]. Frequent worldwide studies reported an increment in the prevalence of depression, anxiety, posttraumatic disorders, substance misuse, and sleep disturbance [8–12]. Unfortunately, most mental health measurement scales are general and not specific to COVID-19. Therefore, a few COVID-19 anxiety-related scales have been invented like the COVID-19 anxiety scale and the fear of COVID-19 scale [13, 14]. Though, some level of infection-related preoccupation is a good motivator for preventive and healthy measure commitment individuals [15]. Though, some level of infection-related preoccupation is a good motivator for preventive and healthy measure commitment [15]. The obsession with COVID-19 scale is a simple brief patient-rated scale that consists of four items with five options rated from zero to four as the following: 0 (Not at all), 1 (rare “less than a day or 2”), 2 (several days), 3 (more than 7 days), and 4 (nearly every day over the last 2 weeks) [15]. The validity and reliability of obsession with COVID-19 scale had been proved through two large adult participants studies in the USA [15]. It has good internal consistency (Cronbach’s alpha > 0.83) and factor analysis addressed the unidirectionality of the scale. Construct validity was accepted when correlated with related scales that include coronavirus anxiety, spiritual crisis, alcohol/drug coping, extreme hopelessness, and suicidal ideation. Scoring equal to or more than seven, or high individual item scores indicates dysfunctional preoccupations that may need more sophisticated evaluation [15]. This scale has been adapted and translated to Urdu, Korean, Spanish, Portuguese, Persian, and Chinese languages [16–22]. All of the newly translated versions addressed good validity and reliability and prove the single factor dimension of the scale which further confirms the original English-version measures [16–22].

We aim in this study to create an Arabic language version of the obsession with COVID-19 scale through professional translation and proper validity and reliability evaluation.

## Methods

We followed a well-known reliable standardized scales translation and adaptation protocol described by Sousa and Rojjanasriratw [23]. Firstly, two bilingual translators translated the scale into Arabic language. Secondly, we developed a unified Arabic version after we compared the two already translated versions. Then, Arabic language linguists reviewed the final version from language perspectives; and minor semantic changes were addressed. After that, two bilingual speakers independently translated back the drafted Arabic scale into English. We synthesized them into one English version and compare them with the original English scale where we did not find a major deviation from the original English scale. Finally, we sent the pre-final Arabic versions to twenty university students and ten faculties to ask their opinion about the clarity of the scale items. All of the items were clear and easy to understand and to be answered. In addition, we asked ten health professionals (psychiatrists, family physicians, and psychologists) to evaluate the scale’s face validity and the appropriateness of the scale to measure the targeted mental health aspects. The obsession with COVID-19 scale is in the public domain where permission is not required for use or translation.

A cross-sectional sample of university students was targeted by an online survey through WhatsApp groups and university emails.

The study was carried out to assess the psychometric properties of the obsession with COVID-19 scale in the Saudi population. The online survey was designed and distributed among students at the University through e-mails and WhatsApp groups. All participants were provided with a plain-language information statement. The poll began with a request for an informed consent declaration, and respondents’ anonymity was ensured. The survey includes some sociodemographic questions, a validated COVID-19 fear scale Arabic version for convergent validity, and the Arabic version of obsession with COVID-19 scale.

From January 10, 2021, through January 25, 2021, data was collected using Google survey forms. The study was approved by the research ethics local committee with the reference number BCOM/H-06-BH-087.

*The obsession with COVID-19 scale* is an effective and valid tool for clinical research and practice, 4-item obsession with COVID-19 scale with strong reliability and validity was considered. This scale was designed and statistically tested in two large independent samples of the population and showed (83% sensitivity and 93% specificity) in discriminating the nonfunctional COVID-19 thinking patterns from those without such pattern [15, 16].

Fear of COVID-19 scale is a 7-item self-reported measure with good item correlation, internal consistency, and reliability [14]. Alyami et al. made and validate the Arabic version of the fear of COVID-19 scale, which had sufficient internal consistency, convergent validity and address two factors by factor analysis as the original English-language version [24].

In statistical analysis, IBM SPSS Statistics Version 20 was used. Cases with missing survey values were not included in the study. The participants’ sociodemographic distribution was also evaluated and described in percentages, with the age presented as the mean standard deviation (SD). Internal consistency was assessed using Cronbach’s alpha, Bartlett’s Test of Sphericity, Kaiser–Meyer–Olkin measure of sample adequacy, and factorial analysis. It was also taken into account the average variance extracted and composite reliability. The relationships between distinct items on the same scale and between the means of multiple scales were investigated using analysis of variance (ANOVA) and Pearson correlation coefficients. An independent *t*-test was also used to measure the differences between scales means and among sociodemographic categories. The Pearson correlation coefficients between various sociodemographic characteristics and the means of scales were addressed. *P* value less than 0.05 considered to be significant.

## Results

We got 253 university students’ responses. Only 233 answers were included while 20 answers were incomplete and not suitable for analysis. Females represented 42.1% of the sample and the rest 57.9% were males, and 55.4% of participants were ≤ 25 years old. Almost one-third of students addressed previous COVID-19 infection with positive screening tests, and 71.7% admitted that at least one of his/her family members had a COVID-19 infection. Table 1 summarized the sociodemographic data of participants.

**Table 1:** Sociodemographic data (no. = 233)

Parameter	No.	Percent	*P* value (*t*-test)
Age (mean ± SD)	25.5 ± 4.41		
Gender			0.154
1. Male	135	57.9%	
2. Female	98	42.1%	
Faculty			0.241
1. Medical	165	70.82%	
2. Non-medical	68	29.18%	
Social status			0.071
1. Married	18	7.73%	
2. Non-married	215	92.27%	
History of chronic disease			0.037
1. Positive	49	21.03%	
2. Negative	184	78.97%	
Previous history of anxiety			< 0.001
1. Yes	97	41.63%	
2. No	136	58.37%	
History of COVID-19 positive test			0.313
1. Yes	71	30.47%	
2. No	162	69.53%	
Family member or colleagues had COVID-19 positive test			< 0.001
1. Yes	167	71.67%	
2. No	66	28.33%	
Family member or colleague death due to COVID-19 infection?			0.082
1. Yes	69	29.61%	
2. No	164	70.39%	

Cronbach’s alpha coefficient of the obsession with COVID-19 scale was 0.82. Item-total correlations ranged between 0.868 and 0.635 while inter-item correlations were between 0.689 and 0.308. Bartlett’s test was significant with *p* value < 0.001, and Kaiser–Meyer–Olkin was 0.744. Factorial analysis extracted one factor that explains 65.33% of the cumulative variances with factor loadings between 0.43 and 0.77. The average Variance Extracted of obsession with COVID-19 scale was 0.65 and composite reliability was (0.88) which is accepted. ANOVA test showed was no significant difference between the means of different items within the scale (*p* value = 0.339). Details regarding the obsession with COVID-19 scale are presented in Table 2.

**Table 2:** Summary of the results of the obsession with COVID-19 scale

Item	Mean ± SD	Factor loadings	Item-total correlation	Inter-item correlations		
				2	3	4
i. …I may have caught the virus	0.777 ± 1.043	0.859	0.868	0.689	0.650	0.381
ii. … certain people I saw may have the virus	0.828 ± 1.116	0.822	0.846		0.607	0.308
iii. I could not stop thinking …	0.751 ± 0.885	0.877	0.858			0.544
iv. I dreamed …	0.670 ± 0.724	0.656	0.635			

Convergent validity was also assessed by correlating the total scores of different scales between obsession with COVID-19 scale and the fear of COVID-19 scale. Generally, they expressed positive correlations in the expected direction which support their validity. The correlation between obsession with COVID-19 scale and fear of COVID-19 scale (*r* = 0.574, *p* < 0.001) was moderately strongly significant.

Independent *t*-test was used to test for sociodemographic significant differences of OCS mean scores. The results of *t*-test were summarized in Table 3.

**Table 3:** Sociodemographic OCS mean differences measured by *t*-test

Sociodemographic information	*P* value
Age (less than 25 year versus more than 25)	0.169
Gender (male versus female)	0.235
Faculty (medical versus non-medical)	0.071
Social state (married versus non-married)	0.204
History of chronic disease (positive versus negative)	0.037
Have you been tested positive COVID-19 infection? (yes, versus no)	0.062
Have you been suffering from anxiety before COVID-19 pandemic? (yes, versus no)	< 0.001
Has any of your family or colleagues tested positive COVID-19 infection? (yes, versus no)	0.0617
Has any of your family or colleagues died due to COVID-19 infection? (yes, versus no)	< 0.001

## Discussion

The translation and adaptation process of obsession with COVID-19 scale followed Sousa and 9 Rojjanasrirat scientific guidelines used in reliable cross-cultural mental health scales translation and adaptation [23], which address cultural particularities and maintain the authentic properties of the scale to be a reliable and valid Arabic version of obsession with COVID-19 scale. Obsessions with the COVID-19 scale Arabic version have good internal consistency (Cronbach’s alpha coefficient 0.82), with item-total correlations ranging between 0.868 and 0.635. Inter-item correlations score between 0.689 and 0.308. These scores are similar to obsession with COVID-19 scale original English version scores (Cronbach’s alpha coefficient 0.83) [15], although higher than some other language validation studies scores (range between 0.71 and 0.74) [16, 18, 22]. Factor analysis test showed one factor that explains 65.33% of the cumulative variances, which proves the unidimensional nature of the scale, which corresponded to similar results in the previous validation studies [16–22]. Moreover, convergent validity is further supported by the moderate correlation between obsession with COVID-19 scale Arabic version and the Arabic version of the COVID-19 fear scale. The ability of the scale to show significant differences between those who had a previous history of anxiety or chronic illnesses and those who do not may further add to the validity of the scale.

There are certain limitations to this study that should be highlighted. The survey’s participants were students from a single university, which may limit the usage of scale by populations with varying sociodemographic characteristics. We also adopted a convenient cross-sectional sample method rather than a random sampling method. Because the study used an electronic survey, persons without an internet connection may not be able to participate. The Arabic version’s cut-off point was yet to be determined. These restrictions, however, do not prevent the questionnaire from being used as they were resolved in the original and several additional language versions.

## Conclusions

The obsession with COVID-19 scale Arabic version is a reliable and valid version that retains the reliability and validity features of the original English version. This version has a good level of internal consistency and convergent validity, making it suitable for assessing dysfunctional obsessions in the context of the COVID-19 pandemic.

## Data Availability

Data are available and will provided upon request.

## Abbreviations

OCS: Obsession with COVID-19 scale
COVID-19: The coronavirus infection disease
SD: Standard deviation
ANOVA: Analysis of variance
SPSS: Statistical Package for the Social Sciences
